# Healthy Eating Index-2015 and Dietary Total Antioxidant Capacity as Predictors of Prediabetes: A Case-Control Study

**DOI:** 10.1155/2021/2742103

**Published:** 2021-07-21

**Authors:** Jamal Rahmani, Karim Parastouei, Maryam Taghdir, Heitor O. Santos, Farinaz Hosseini Balam, Mohsen Saberi Isfeedvajani

**Affiliations:** ^1^Health Research Center, Life Style Institute, Baqiyatallah University of Medical Sciences, Tehran, Iran; ^2^School of Medicine, Federal University of Uberlandia (UFU), Uberlandia, Minas Gerais, Brazil; ^3^Department of Cellular and Molecular Nutrition, School of Nutrition Sciences & Food Technology, Shahid Beheshti University of Medical Sciences, Tehran, Iran; ^4^Medicine, Quran and Hadith Research Center & Department of Community Medicine, Faculty of Medicine, Baqiyatallah University of Medical Sciences, Tehran, Iran

## Abstract

**Background:**

The overall dietary quality, as well as the dietary total antioxidant capacity (DTAC), deserves central attention in the management of borderline high glucose levels since nonpharmacological strategies are imperative in this regard. Thus, we aimed to investigate the association between prediabetes with dietary quality and DTAC.

**Methods:**

A case-control study was conducted on 49 patients with prediabetes and 98 controls. Demographics, anthropometric measures, and fasting blood glucose levels of all participants were obtained. Participants completed a validated 80-item food frequency questionnaire (FFQ). DTAC scores were generated using FFQ data, and Healthy Eating Index-2015 (HEI-2015) was used as a diet quality index. The lowest tertile of HEI-2015 and DTAC was considered as the reference category, and logistic regression was used to estimate the relationship between prediabetes with HEI-215 and DTAC.

**Results:**

Mean age and body mass index of participants were 47.42 ± 15.98 years and 27.90 ± 4.96 kg/m^2^. Patients with prediabetes had lower DTAC scores when compared to controls (11.86 ± 5.77 and 17.81 ± 12.08, *P* = 0.01). There was a significant inverse association between the highest tertile of the DTAC score when compared with the lowest tertile in crude (OR = 0.11; 95% CI: 0.03–0.43), age-adjusted (OR = 0.13; 95% CI: 0.03–0.48), and fully adjusted (OR = 0.09; 95% CI: 0.02–0.53) models. In contrast, there was no difference between HEI-2015 in patients with prediabetes when compared to controls (74.41 ± 8.91 and 74.41 ± 9.35, respectively; *P* = 0.85). Correspondingly, no difference was observed between the highest tertile of the HEI-2015 score when compared with the lowest tertile in crude (OR = 1.23; 95% CI: 0.53–2.86), age-adjusted (OR = 1.17; 95% CI: 0.48–2.82), and fully adjusted (OR = 1.53; 95% CI: 0.56–4.16) models.

**Conclusion:**

This study demonstrates a clear association between prediabetes with less DTAC, but not with HEI-2015.

## 1. Introduction

Oxidative stress plays an substantial role in the pathogenesis of diabetes and its complications, including diabetic neuropathy, nephropathy, retinopathy, and macrovascular complications, among a multitude of related problems [1, 2]. At the most basic level, oxidative stress caused by redox imbalance impairs signaling pathways involved in *β*-cell function and insulin sensitivity mainly via reactive oxygen species [3]. More importantly, diabetes remains a burden of public health worldwide; as such, in 2019, almost half a billion people (463 million people) around the world suffered from diabetes, whereas the prevalence is estimated to rise by 25% in 2030 and 51% in 2045 [4]. The prevalence of prediabetes has also been increasing with a forecast of >470 million people with prediabetes in 2030 [5]. According to the American Diabetes Association (ADA), prediabetes is classified as 100–125 mg/dL fasting blood glucose (FPG), 140–199 mg/dL 2 h postprandial blood glucose, or HbA1c 5.7–6.4%, whereby a massive effort based on pharmacological and nonpharmacological interventions is deemed imperative to control these biomarkers [6].

Taken together, the treatment of diabetes consists of diet modification, body weight loss, physical exercise, oral medications, and insulin therapy [7], in which lifestyle changes are the cornerstone for reversing prediabetes and are capable of reducing the relative risk of progression to diabetes by 40%–70% [5]. Given the relevance of nonpharmacological strategies against prediabetes, one must be mindful of the need to ameliorate the diet quality based on a constellation of popular food groups alongside the dietary antioxidants as a myriad of essential (e.g., magnesium, zinc, chromium, calcium, potassium, and vitamin D) and functional (e.g., flavonoids and phenolic acids) nutrients can improve glucose metabolism [8–12] or even attenuate glucose absorption, a role played by fiber intake [13].

Healthy Eating Index (HEI) is a measure used to assess the overall diet quality, monitoring the compliance between a set of foodstuffs with central recommendations of the Dietary Guidelines for Americans [14]. The HEI was originally developed in 1995, and HEI-2015 is the most current version and has been adopted by the population worldwide [15]. The dietary total antioxidant capacity (DTAC), in turn, is a more specific measure to estimate an adequate intake of dietary antioxidants. Along these lines, we carried out a case-control study using the DTAC and HEI-2015 to investigate the potential of overall dietary quality and antioxidants in forecasting prediabetes.

## 2. Methods

### 2.1. Subjects

This is a study with a hospital-based case-control design. The participants were recruited from Baqiyatallah Hospital in Tehran, Iran, from January to March 2021. Cases with recently diagnosed (<6 months) prediabetes and healthy controls aged 18–90 years were invited to participate. Participation in this study was voluntary. FBG was measured after an overnight fast (at least 8 h), with values between 100 and 125 mg/dL being detected <6 months before the interview and confirmed by a physician as part of inclusion criteria for the cases, whereas the control group had FBG <100 mg/dL. The control group involved healthy men and women without any related metabolic diseases. Each participant was interviewed face to face. The height of participants was measured with a nonstretchable stadiometer (model Seca 206) to the nearest 0.1 cm, and the body weight was measured on a digital scale (model Seca 803) with an accuracy of 0.1 kg with a minimal amount of clothing and no shoes. All interviews and measurements were done with trained medical staff. We collected information about age, gender, weight, height, marital status, education levels, occupation, income, physical activity, dietary supplementation (any supplement brands and related products), and family history of diabetes of participants. Body mass index (BMI, kg/m^2^) was calculated using body weight and height. We excluded patients with diabetes, pregnant or lactating women, and those on a special diet during the previous year of the study. This research was approved by the Ethics Committee of Baqiyatallah University of Medical Sciences (IR.BMSU.REC.1399.287), and all participants signed a consent form before participation.

### 2.2. Diet Assessment

A semiquantitative valid and reproducible food frequency questionnaire (FFQ) containing 80 food items was applied by a registered dietitian to collect typical dietary information of participants. Frequency of each food item was obtained, and then the information was transformed into an average daily intake in the FFQ. Food energy and macro- and micronutrient contents were determined with modified Nutritionist IV for Iranian foods (version 4.1, 1997; First Databank, Hearst Corporation, San Bruno, CA).

HEI-2015 includes 13 components as follows: 1-total fruits (whole fruits and fruit juice), 2-whole fruits, 3-total vegetables (legumes (beans and peas), dark-green vegetables, and all other vegetables), 4-greens and beans, 5-total protein foods (meat, poultry, eggs, seafood, nuts, seeds, legumes (beans and peas), and soy products), 6-seafood and plant proteins (seafood, nuts, seeds, legumes (beans and peas), and soy products), 7-whole grains (legumes (beans and peas) and dark-green vegetables), 8-dairy, 9-fatty acids, 10-refined grains, 11-sodium, 12-added sugars, and 13-saturated fats (ratio of polyunsaturated and monounsaturated fatty acids to saturated fatty acids) [16]. Five points were considered for each of the following items: total fruits, whole fruits, total vegetables, greens and beans, total protein foods, and seafood and plant proteins, and other items have ten points. Each food group in the FFQ was translated to cup and ounce equivalents.

Two items of HEI-2015 (added sugars and saturated fats) were converted into percentage of total calorie intake to represent the dietary intake of foods and nutrients by density as amounts per 1000 kcal of intake except for fatty acids. The HEI-2015 score ranged from 0 to 100.

For calculating DTAC, the ferric reducing-antioxidant power (FRAP) values were used [17]. The FRAP assay measures the ability of DTAC in reducing ferric to ferrous ions, which is reported as mmol/100 grams of foods [18]. We assigned the nearest comparable food value for food items without TAC data. FRAP values of food items were multiplied by the food consumption volume and summed up to obtain the DTAC for each participant.

### 2.3. Statistical Analyses

Independent two-tailed *T*-test and *χ*^2^ analysis were used to evaluate the demographic distribution and nutrient intake in control and case groups. Bivariate correlations were used to assess the relationship between BMI with HEI-2015 and DTAC. The overall HEI-2015 and DTAC scores were categorized into tertiles based on their distribution among control participants. Logistic regression analysis was undertaken followed by adjustment for potential confounding variables, including age, gender, BMI, marital status, education levels, occupation, income, physical activity, dietary supplementation, family history of diabetes of participants, and total calorie intake. The lowest tertile of HEI-2015 and DTAC was taken to be as the reference category to calculate odds ratios (ORs) with 95% confidence intervals (95% CIs). SPSS (v23, IBM Corp.) was used for all statistical analyses with significance accepted at *α* = 0.05.

## 3. Results

Information from 147 participants was collected, whereby 49 were recently diagnosed with prediabetes (<6 months) and 98 constituted healthy controls. The characteristics of the participants are provided in Table 1. The mean age and BMI of participants were 47.42 ± 15.98 years and 27.90 ± 4.96 kg/m^2^, respectively. The family history of diabetes was not significant between the case and control groups (*P* = 0.52), reaching zero in the latter. Dietary supplements were used in 20.4% and 22.9% of the participants in the control and prediabetes groups, respectively. The education levels of the control group were higher than those of the prediabetes group (*P* = 0.04). In the control group, 32.7%, 41.8%, and 25.5% of the participants had normal weight, overweight, and obesity, respectively, and 28.6%, 34.7%, and 36.7% in the prediabetes group (*P* = 0.36). There was no significant correlation between HEI and BMI (*r* = −0.04 and *P* = 0.59) and between DTAC and BMI (*r* = 0.01 and *P* = 0.86).

Table 2 shows the average micro- and macronutrient intakes for each group. The mean total calorie intake was 1930.28 ± 1125.14 kcal/d in cases and 2554.78 ± 1794.70 kcal/d in controls (*P* = 0.01, difference between groups). Total protein and fat intake had no significant difference between the two groups, but the control group consumed more carbohydrates (376.79 ± 280.45 g/day vs. 252.94 ± 147.65 g/day; *P* = 0.01) and fiber (10.58 ± 9.49 g/day vs. 7.64 ± 4.83 g/day; *P* = 0.01) compared to the prediabetes group.

Patients with prediabetes had lower DTAC scores when compared to controls (11.86 ± 5.77 and 17.81 ± 12.08, *P* = 0.01). However, there was no difference between HEI-2015 in patients with prediabetes when compared to controls (74.41 ± 8.91 and 74.41 ± 9.35, respectively; *P* = 0.85).

Cases and controls were classified into tertile categories based on their HEI-2015 and DTAC scores. The cutoff for each category was as follows: HEI-2015: *T*1 ≤ 71.23, *T*2: 71.23–78.21, and *T*3 > 78.21 and DTAC: *T*1 ≤ 11.90, T2: 11.90–21.24, and *T*3 > 21.24. The ORs for prediabetes based on its association with HEI-2015 and DTAC tertiles are derived in Table 3, with *T*1 considered as the reference category (i.e., OR = 1).

The OR for prediabetes in the highest tertile of the HEI-2015 score was not different from the lowest tertile in the crude (OR = 1.23; 95% CI: 0.53–2.86) and age-adjusted (OR = 1.17; 95% CI: 0.48–2.82) models. The association between HEI-2015 and prediabetes remained nonsignificant in the fully adjusted model (OR = 1.53; 95% CI: 0.56–4.16) (Table 3).

In the highest tertile of the DTAC score, a significant inverse OR for prediabetes was noted in the crude (OR = 0.11; 95% CI: 0.03–0.43) and age-adjusted (OR = 0.13; 95% CI: 0.03–0.48) models. Moreover, this association remained significant in the fully adjusted model (OR = 0.09; 95% CI: 0.02–0.53) (Table 3).

Logistic regression-derived OR and 95% CIs for adjusted confounders associated with prediabetes are provided in Supplementary Table 1. Increasing age showed a direct association with prediabetes (OR = 1.04; 95% CI: 1.01–1.08), but the association was nonsignificant in overweight participants (OR = 1.01; 95% CI: 0.36–2.79) and in obese participants (OR = 1.64; 95% CI: 0.57–4.67) compared to normal weight.

## 4. Discussion

We carried out a case-control study to ascertain the impact of HEI-2015 and DTAC scores as predictors of prediabetes. There was a significant inverse association between the highest tertile of the DTAC score when compared with the lowest tertile in both crude and adjusted models. More specifically, the likelihood of having prediabetes was 89% (OR = 0.11; 95% CI: 0.03–0.43), 87% (OR = 0.13; 95% CI: 0.03–0.48), and 91% (OR = 0.09; 95% CI: 0.02–0.53) lower in the crude model, age-adjusted model, and fully adjusted model, respectively. On the contrary, there was no difference between the tertiles of HEI-2015 scores neither in crude nor in adjusted models.

Regarding the characteristics of the participants, in addition to the already expected higher FBG, case patients were older when compared to controls. This was the main reason that led us to perform the age-adjusted model. Furthermore, there was a lower intake of total calories, total carbohydrate, vitamin C, B6, and B9, and zinc in patients with prediabetes compared to controls. Insofar as the total calorie intake may be regarded as the main element of this list, we added it together with crucial confounding factors to the fully adjusted model (i.e., adjustment for age, gender, BMI, marital status, income, occupation, education, physical activity, dietary supplementation, total calorie intake, and family history of diabetes). Albeit a higher intake of total calorie intake and carbohydrates is generally expected in patients with prediabetes or related cardiometabolic disorders, the inverse association that we found can be justified by underreporting, which unfortunately is a typical problem in nutrition research [19].

Beyond macronutrients and calories, the intake of antioxidants has gained vast attention to global public health, which are nonenzymatic elements classified primarily as flavonoids, minerals, vitamins, and derivatives [20–23]. In this sense, several nutraceutical agents (e.g., some herbal medicines and supplements) and functional foods have been proposed in the management of glucose levels due to their antioxidant features [24–29]. Nevertheless, the antioxidant potential across the diet overwhelms those adjunctive strategies since long-term adherence is a cornerstone, in which hallmark foodstuffs such as fruits, vegetables, seeds, whole grains, oils, and particular beverages (e.g., coffee and tea) must be endorsed [30, 31]. Bearing this concept in mind, our study contributes to the landscape of patients with borderline high glucose levels.

Unlike macronutrients, counting antioxidant intake remains a challenge in the real-world prescription-based intervention, but some questionnaire-based measures have emerged in an attempt to forecast antioxidant compounds among personalized eating plans. Herein, we used HEI-2015 to assess the diet quality, which is a measure used to monitor the compliance between a set of foodstuffs with central recommendations of the Dietary Guidelines for Americans and which has been extrapolated to other populations so far [14]. In addition, considering that HEI-2015 is insufficient to assess dietary antioxidants, we calculated the DTAC of foods using FRAP, which is a viable indicator to guide the impact of dietary antioxidants on glucose metabolism [32]. Correspondingly, DTCA is strongly associated with a plethora of dietary antioxidants (e.g., carotenoids, *β*-carotene, *β*-cryptoxanthin, flavonoids, isoflavones, flavan-3-ols, flavones, and flavonols) and circulating biomarkers of antioxidant status (e.g., TAC, glutathione peroxidase, red blood cell glutathione peroxidase, *α*-tocopherol, and lutein levels) in both sexes [33]. Additionally, DTAC showed positive and significant associations with fiber, folic acid, vitamins A and C, magnesium, selenium, and zinc intakes in patients with metabolic syndrome even when adjusted for sex and daily energy intake [34]. In light of this context, the present research adds to the literature fundamental results concerning the use of DTCA with a focus on prediabetes.

Previous research demonstrates that DTAC is negatively associated with some components of the metabolic syndrome, such as FBG, regardless of sex and daily energy intake [34]. Likewise, we observed a negative association between high DTAC score and prediabetes through a statistical adjustment model that combined sex and daily energy intake. It is crucial to highlight that DTAC has been investigated in many diseases and metabolic disorders, whereby elevated FBG is a related concern. For instance, in a systematic review of observational studies, there was a substantial inverse association between high DTAC and FBG along with other CVD-related risk factors, such as C-reactive protein, blood pressure, and waist circumference, with a positive association for high-density lipoprotein cholesterol as well [35]. Another systematic review of observational studies showed that higher DTAC may have protective effects against cancer (e.g., colorectal, gastric, and endometrial cancer) [36], while epidemiologic evidence suggests that patients with higher FBG levels are at significantly higher risk for a variety of cancers [37, 38].

It must be noted that this research was performed in a developing country, and the burden of diabetes, both in terms of prevalence and number of adults affected, has grown more intensively in low-income and middle-income countries than in high-income countries [39]. In addition, other studies conducted in Iran used DTAC as a predictor of cardiometabolic dysregulation. For instance, in a study enrolling 1938 Iranian patients, after a 3-year follow-up, those with the highest DTAC score had a lower likelihood of having metabolic syndrome, as well as abdominal obesity and hypertension [40]. In a cross-sectional study consisting of 454 hospitalized Iranian candidates for coronary artery bypass graft surgery, in turn, higher DTAC was associated with a lower prevalence of hypertension, lower hematocrit, lower total cholesterol, and higher albumin and vitamin D concentrations [41]. Therefore, DTAC can be considered a feasible and cost-effective approach for investigating hospitalized patients.

Our study has some limitations that ought to be mentioned. First of all, the case-control design does not allow inferring causation or recommendation, but it is at least useful to verify the prognosis of diseases, which sharply cannot be ruled out. Secondly, taking into consideration an epidemiological basis, the sample size was limited by comprising only one population (Tehran, Iran) and, therefore, may not be representative of other populations and countries. Thirdly, the use of FFQ may be questionable, but it is a recognizable tool in nutrition research [42]. Another limitation of the study was the lack of a structured questionnaire to collect physical activity information. Ultimately, we did not measure biomarkers of oxidative stress and antioxidant status due to obstacles caused by the COVID-19 pandemic. In the face of all limitations, our data are noteworthy, given the promising effects of DTAC against oxidative damage and related clinical complications caused by higher glucose levels [34]. Furthermore, to the best of our knowledge, this is the first work that investigated the association between HEI-2015 and prediabetes.

## 5. Conclusion

This case-control study found that high DTAC is associated with a significantly reduced likelihood of having prediabetes. However, no significant association was observed between HEI-2015 and prediabetes. Further longitudinal studies with larger and different populations, employing pragmatic or promising biomarkers of oxidative stress and antioxidant status at the same time, are warranted to expand our findings.

## Figures and Tables

**Table 1:** Sociodemographic characteristics of participants^a^.

Characteristic	Controls (*n* = 98)	Cases (*n* = 49)	*P* value^b^
Age	44.08 ± 15.98	54.10 ± 13.89	0.01
BMI^c^	27.81 ± 4.89	28.08 ± 5.11	0.76
FBG^d^	88.14 ± 6.93	118.10 ± 18.27	0.01

Gender			
Female	54 (55.1)	23 (46.9)	0.47
Male	44 (44.9)	26 (53.1)	

Educational status			
Primary or less	14 (14.3)	13 (26.5)	0.04
Secondary/high school	36 (36.7)	22 (44.9)	
Tertiary/university	48 (49.0)	14 (28.6)	

Marital status			
Single	21 (21.4)	4 (8.2)	0.05
Married	71 (72.4)	44 (89.8)	
Widowed/divorced	6 (6.2)	1 (2.0)	

Occupation			
Employed	60 (61.2)	34 (69.4)	0.21
Unemployed	38 (38.8)	15 (30.6)	

Income			
Low	52 (53.1)	27 (55.2)	0.29
Moderate	32 (32.7)	11 (22.4)	
High	14 (14.2)	11 (22.4)	

Obesity			
Normal	32 (32.7)	14 (28.6)	0.36
Overweight	41 (41.8)	17 (34.7)	
Obese	25 (25.5)	18 (36.7)	
Physical activity (yes)	39 (39.8)	19 (38.8)	0.52
Supplementation (yes)	20 (20.4)	11 (22.9)	0.44
Family history of diabetes (yes)	46 (46.9)	22 (45.8)	0.52

^a^Data are presented as *n* (%) or mean ± SD. ^b^Distribution of continuous variables assessed with independent *t*-test and categorical variables with chi-square test. ^c^Body mass index. ^d^Fasting blood glucose.

**Table 2:** Distribution of macro- and micronutrient intakes across case and control participants^a^.

Characteristic	Controls (*n* = 98)	Cases (*n* = 49)	*P* value^b^
Total calories (kcal/day)	2554.78 ± 1794.70	1930.28 ± 1125.14	0.01
Total protein intake (g/day)	83.55 ± 59.36	70.76 ± 42.40	0.18
Total carbohydrate intake (g/day)	376.79 ± 280.45	252.94 ± 147.65	0.01
Total fat intake (g/day)	88.29 ± 70.47	76.49 ± 58.48	0.31
SFA^c^ (g/day)	20.29 ± 17.32	17.21 ± 11.67	0.20
MUFA^d^ (g/day)	27.57 ± 22.88	23.88 ± 21.33	0.34
PUFA^e^ (g/day)	28.64 ± 27.09	24.47 ± 21.71	0.05
Vitamin A (RAE/day)	2993.67 ± 3673.42	2525.70 ± 2155.23	0.41
Vitamin D (ug/day)	2.32 ± 3.42	2.23 ± 3.62	0.88
Vitamin E (mg/day)	20.37 ± 19.08	16.16 ± 15.19	0.15
Vitamin C (mg/day)	394.26 ± 433.56	214.65 ± 159.27	0.01
Vitamin B6 (mg/day)	2.40 ± 2.14	1.66 ± 1.02	0.01
Vitamin B9 (ug/day)	520.85 ± 467.31	392.60 ± 286.67	0.04
Vitamin B12 (ug/day)	6.93 ± 11.67	4.84 ± 7.60	0.25
Zinc (mg/day)	11.32 ± 8.11	8.94 ± 5.12	0.03
Iron (mg/day)	16.55 ± 12.13	13.26 ± 10.68	0.09
Calcium (mg/day)	1199.77 ± 925.31	966.01 ± 681.59	0.11
Fiber (g/day)	10.58 ± 9.49	7.64 ± 4.83	0.01

^a^Data are presented as mean ± SD. ^b^Independent sample *t*-test was used for continuous variables. ^c^Saturated fatty acids. ^d^Monounsaturated fatty acids. ^e^Polyunsaturated fatty acids.

**Table 3:** Odds ratios and confidence intervals for the association between Healthy Eating Index-2015 and dietary total antioxidant capacity with prediabetes.

Control/case	Tertile 1	Tertile 2	Tertile 3	*P* value for trend
*Healthy Eating Index-2015*				
	33/15 ≤71.23	33/16 71.23–78.21	32/18 >78.21	
Crude	Ref	1.06 (0.45–2.50)	1.23 (0.53–2.86)	0.61
Model 1^a^	Ref	1.06 (0.43–1.59)	1.17 (0.48–2.82)	0.72
Model 2^b^	Ref	1.33 (0.48–3.67)	1.53 (0.56–4.16)	0.40

*Dietary total antioxidant capacity (DTAC)*				
	33/26 ≤11.90	33/20 11.90–21.24	32/3 >21.24	
Crude	Ref	0.76 (0.36–1.63)	0.11 (0.03–0.43)	0.01
Model 1^a^	Ref	0.68 (0.30–1.51)	0.13 (0.03–0.48)	0.01
Model 2^b^	Ref	0.69 (0.28–1.70)	0.09 (0.02–0.53)	0.01

^a^Adjusted for age. ^b^Adjusted for age, gender, BMI, marital status, income, occupation, education, physical activity, dietary supplementation, family history of diabetes, and total calorie intake.

## Data Availability

The datasets used to support the findings of this study are available from the corresponding author upon reasonable request.

## Additional Points

*Highlights*. Higher DTAC scores were inversely associated with prediabetes. Personalized strategies are crucial to translate the DTAC scores into the clinical practice. Higher HEI-2015 scores were not associated with prediabetes.

## Supplementary Materials

Supplementary Materials Supplementary Table 1: logistic regression-derived odds ratios and 95% confidence intervals for adjusted confounders associated with prediabetes. *s*
