# Supplementary material for: Healthy Eating Index-2015 and Dietary Total Antioxidant Capacity as Predictors of Prediabetes: A Case-Control Study
Source: Int J Endocrinol. 2021 Jul 21;2021:2742103. doi: 10.1155/2021/2742103 (PMC8318758; doi:10.1155/2021/2742103)
Supplement: Supplementary Materials — Supplementary Table 1: logistic regression-derived odds ratios and 95% confidence intervals for adjusted confounders associated with prediabetes. s [file 2742103.f1.docx]

Supplement table 1. Logistic regression-derived Odds ratios and 95% confidence intervals for adjusted confounders associated with prediabetes

| Variables | | Exp(B) | 95% C.I.for EXP(B) | |
| --- | --- | --- | --- | --- |
|  |  |  | Lower | Upper |
| Age | Age | 1.04 | 1.01 | 1.08 |
| Gender | Male | 1 | 1 | 1 |
|  | Female | 1.46 | 1.16 | 1.33 |
| Obesity | Normal | 1 | 1 | 1 |
|  | overweight | 1.01 | 0.36 | 2.79 |
|  | Obese | 1.64 | 0.57 | 4.67 |
| Physical activity | No | 1 | 1 | 1 |
|  | Yes | 1.35 | 0.58 | 3.13 |
| Marital status | Single | 1 | 1 | 1 |
|  | Married | 1.39 | 0.30 | 6.42 |
|  | Widowed / divorced | 0.31 | 0.01 | 5.46 |
| Education level | Primary or less | 1 | 1 | 1 |
|  | Secondary / High School | 0.75 | 0.23 | 2.48 |
|  | Tertiary / University | 0.45 | 0.10 | 2.02 |
| Income | Low | 1 | 1 | 1 |
|  | Moderate | 0.61 | 0.21 | 1.79 |
|  | High | 1.34 | 0.40 | 4.25 |
| Occupation | Unemployed | 1 | 1 | 1 |
|  | Employed | 1.40 | 0.47 | 4.14 |
| Dietary supplementation | No | 1 | 1 | 1 |
|  | Yes | 1.29 | 0.47 | 3.51 |
| Family history of diabetes | No | 1 | 1 | 1 |
|  | Yes | 1.32 | 0.57 | 3.04 |
| Total calories intake | total calories intake | 1.00 | 0.99 | 1.01 |
